# IgE‐mediated lipid transfer protein allergy in children

**DOI:** 10.1111/pai.70064

**Published:** 2025-03-24

**Authors:** Bianca Olivieri, Mattia Giovannini, Benedetta Pessina, George Du Toit, Simona Barni, Patrizia Bonadonna, Marco Caminati, Ru‐Xin Foong, Francesca Mori, Elio Novembre, Gianenrico Senna, Isabel Skypala

**Affiliations:** ^1^ Allergy Unit Verona University Hospital Verona Italy; ^2^ Department of Health Sciences University of Florence Florence Italy; ^3^ Allergy Unit Meyer Children's Hospital IRCCS Florence Italy; ^4^ Department of Women and Children's Health (Paediatric Allergy), School of Life Course Sciences, Faculty of Life Sciences and Medicine King's College London London UK; ^5^ Children's Allergy Service Evelina London Children's Hospital, Guy's and St Thomas' Hospital London UK; ^6^ Department of Medicine University of Verona Verona Italy; ^7^ Department of Allergy & Clinical Immunology Royal Brompton & Harefield Hospitals, Part of Guys and St Thomas NHS Foundation Trust London UK; ^8^ Department of Inflammation and Repair Imperial College London UK

**Keywords:** children, food allergy, IgE, lipid transfer protein, LTP, Pru p 3

## Abstract

Lipid Transfer Protein (LTP) allergy, traditionally more prevalent in adults from Southern Europe, is increasingly recognized in pediatric populations worldwide. This review explores the epidemiology, pathogenesis, clinical manifestations, diagnosis, and management of LTP allergy in children. LTP allergy can present with severe systemic symptoms both in children and adults; in children‐only studies, anaphylaxis is reported in up to half of the patients. Moreover, children often display polysensitization to multiple plant‐based foods. The prevalence of LTP allergy among children remains under‐researched, contributing to diagnostic and clinical practice variability. Key allergenic sources involved include peach (Pru p 3) and other *Rosaceae* fruits, as well as tree nuts, with cofactors such as physical activity frequently triggering or exacerbating reactions. Advancements in understanding natural tolerance and targeted therapies, along with expanding LTP immunotherapy, offer promising directions for improving the management of this challenging condition in pediatric patients.

**Key message**

Lipid transfer protein allergy in children is an under‐researched but common entity in European countries. Children often show severe reactions, polysensitization to *Rosaceae* fruits and nuts, and difficulties in recognizing cofactors. However, this age group may be the one who benefits the most from interventions that induce or promote food tolerance. Advancements in understanding LTP allergy clinical spectrum, diagnostic algorithms, and therapeutic management in this specific group of patients will contribute to target and ameliorate these young patients' care.

## 1 INTRODUCTION

Lipid Transfer Protein (LTP) allergy is typically more prevalent in the Mediterranean area, but is becoming increasingly common in Northern Europe. LTP is a pan allergen present in a wide range of foods of plant origin, such as fruits, vegetables, nuts, legumes, and cereals. Sensitization to LTP may be asymptomatic, but when it leads to reactions involving foods, it is referred to as LTP allergy. If reactions involve foods from multiple, taxonomically unrelated groups, it is classified as LTP syndrome. LTP allergy presents with clinical manifestations of varying severity and often involves cofactors, making the management of this allergy even more complex.^1^, ^2^

Whilst LTP allergy is well characterized in adults, it is less well understood in children, so it may remain underdiagnosed, despite LTP being a common sensitizing allergen.^3^, ^4^ In a Spanish cohort, sensitization to Pru p 3, the peach LTP often used as a marker of LTP sensitization, was twice as high in children compared with adults, 22% versus 11%, respectively.^5^ It is believed that LTP sensitization often occurs early in life.^6^, ^7^, ^8^ In a recent study on 26 children with LTP allergy, more than 50% reported the first reaction before 12 years of age, while 12% had the first reaction before 3 years of age.^9^ In contrast, an Italian study reported that the level of sensitization to Pru p 3 peaks around the beginning of the third decade of life.^10^ Another study showed that when allergy to peach begins in early childhood, it is likely linked to a sensitization to Pru p 3, with earlier onset correlating with higher IgE levels. Additionally, children sensitized to Pru p 3 tend to exhibit clinical manifestations sooner than those sensitized to pollen‐related allergens.^11^

This review delves into the epidemiology, pathogenesis, clinical features, diagnosis, and management of LTP allergies in children, acknowledging this condition's complexity and rising prevalence. Table 1 summarizes the main studies on LTP allergy involving pediatric populations.

**TABLE 1:** Summary of main studies involving LTP pediatric allergic patients.

Study (Author, journal, year)	Type of study	Country	Population (*n* and demography)	Food triggers	Sensitization profile	Clinical manifestations	Cofactors	Notes
Pediatric studies								
Muñoz‐Osores E et al. *Ann Allergy Asthma Immunol* 2023	Retrospective observational Children only	Chile	*N* = 32 F 34% Median age 7 y (0–16) All with LTP allergy	53.1% non‐*Rosaceae* family fruits 43.8% *Rosaceae* family fruits 53.1% tree nuts 53.1% legumes 15.6% vegetables 3.1% seeds	Co‐sensitization: 33.3% profilin 0% PR‐10	66% OAS 53% anaphylaxis 25% U/AE 9% GI symptoms	2 Exercise	LTP syndrome in 75% Higher frequency of allergic comorbidities (91% vs. 61%) and anaphylaxis (53% vs. 30%–40%) compared to Europe All 7 patients with Pru p 3 and profilins co‐sensitization had anaphylaxis upon consumption of LTP‐containing foods
Pascal M et al. *Allergol Immunopathol* (*Madr*) 2016	Retrospective observational Children only	Spain	*N* = 130 F 36.2% Mean age 10.8 y (3–18)	69.2% ≥2 unrelated plant‐foods 13.1% stone‐fruits only 14.6% many taxonomically‐related nuts, seeds or legumes 3.1% kiwi Most common foods: peach, walnut, hazelnut, and peanut	83.1% Pru p 3+ 77.7% Jug r 3+ 56.2% Ara h 9+ 55.4% Cor a 8+ 26.2% Tri a 14+ 69.3% pollen LTPs+ Co‐sensitization: 65.4% storage‐proteins 18.5% profilin 12.3% PR‐10	45.4% anaphylaxis 42.3% U/AE	5 Exercise (in 3 patients)	Anaphylaxis was more frequent: with nuts/seeds/legumes vs. fruit/vegetables (*p* < .05) with co‐sensitization with storage proteins Specific IgE levels to LTPs did not correlate with reaction severity Peach tolerance in 69% of Pru p 3 positive subjects, walnut tolerance in 63% of Jug r 3 positive subjects
Barradas Lopes J et al. *Eur Ann Allergy Clin Immunol* 2023	Retrospective observational Children only	Portugal	*N* = 26 F 50% Median age 10 y (1–17)	69% multiple foods 69% fruits (62% peach) 50% tree nuts 8% peanut 4% sesame	100% Pru p 3 73% Jug r 3 35% Cor a 8 Co‐sensitization: 15% (2 profilin and 2 PR‐10)	58% U 46% anaphylaxis 42% OAS	6 Exercise 1 NSAIDs	No association between severe reactions and wheal SPT to LTP extract/sIgE to Pru p 3 or Cor a 8/co‐sensitization pattern 12% reported reactions to new LTP containing foods during follow‐up 38% tolerate fruits/tree nuts for which they are sensitized 92% tolerate *Rosaceae* family fruits without peel
Ciprandi G et al. *Acta Biomed* 2019	Retrospective observational Children compared to adults	Italy	*N* = 82 F 33% Mean age 8.19 ± 4.23 y Allergic rhinitis to Parietaria + sensitization to Pru p 3	36.6% peach 25.6% walnut 15.8% apple		30.0% anaphylaxis 29.3% other systemic symptom 26.7% OAS		Reactions to LTP foods were more common in children vs. adults (73.2% vs. 37.3%, *p* = .0007) Pru p 3 levels were higher in children vs. adults (*p* = .042)
Boyano‐Martínez T et al. *Pediatr Allergy Immunol* 2013	Prospective cross‐sectional Children only	Spain	*N* = 57 F 43.9% Mean age 7.4 y (2–17) All with peach allergy	56% reported reactions with LTP‐kazacontaining foods: 43.9% other fruits 29.8% peanut or nuts 8.8% legumes	96% Pru p 3 Co‐sensitization: 11% Pru p 1 10% Pru p 4	95% mucocutaneous 26% systemic reaction 21% respiratory 18% vomiting		OFC with peach pulp was negative in 52/56 (93%)
Kazancioglu A et al. *Allergy Asthma Proc* 2024	Retrospective observational Children only	Turkey	*N* = 105 F 22.9% Median age 5 y (3–8)	*N* = 59 (56.2%) clinically reactive to LTP foods	74% Pru p 3 66% Cor a 8 60% Mal d 3	43% systemic reaction 18% anaphylaxis 11% OAS		Median n of LTP allergen molecule positivity was higher in clinically reactive (*p* < .01) and was associated with risk of anaphylaxis (OR 1.315 *p* = .01) 69.4% of clinically reactive were also sensitized to storage proteins
Children and adult studies								
Faber MA et al. *J Allergy Clin Immunol* 2017	Retrospective observational Children and adults	Belgium	*N* = 718 with pollinosis/plant food allergy ➔ *N* = 177 LTP+ (*N* = 90 <18 y) 3 groups: pollen allergic PFA pollen + PFA		86% Pru p 3 73% Mal d 3 79% Bet v 1+			Most patients tolerate culprit food (tolerance in 35%–59% of sensitized patients) No differences in LTP sIgE reactivity between clinical phenotypes/sexes/age groups (preschool‐schoolchildren‐adults) BAT predictive of clinical reaction vs. tolerance to peach
Scala E et al. *Allergy* 2015	Retrospective observational Children and adults	Italy	*N* = 568 F 54.6% Mean age 27.5 ± 16.2 y (1–84)		82% Pru p 3 71% Jug r 3 50% Ara h 9 27% recognized only 1/7 tested LTPs (45% Pru p 3) Co‐sensitization: 23% PR‐10 18% profilin 7% both	34.3% L + S + R 12.9% S + R 12.7% R 12.1% L + S 9.3% S 7.6% L + R 6.9% L 4.2% other		IgE reactivity to Pru p 3 was more prevalent (94%) among children <6 y (94%) Recognition of >5 LTPs molecules was linked with an increased risk of SR (*p* < .001) Co‐sensitization to Par j 2 and PR‐10 or profilin associated with a lower prior prevalence of severe food‐induced reactions
Scala E et al. *Eur Ann Allergy Clin Immunol* 2023	Retrospective observational Children and adults	Italy	*N* = 426 F 56.1% Mean age 34 ± 16 y (2–74)		77% Pru p 3 60% Mal d 3 60% Zea m 14 50% Ara h 9 6% Tri a 14 50% sensitized to >5 LTPs Co‐sensitization: 27.7% PR‐10 13.1% profilin 5.4% polcalcin	65.8% moderate reaction 34.2% severe reaction 34% OAS 18.8% tolerant to LTP‐containing foods	46 (10.8%) Exercise 53 (12.4%) NSAIDs	Increased risk of severe reaction if: higher specific IgE sensitization to >5 molecules presence of cofactor positivity to Ara h 9, Cor a 8, Mal d 3 Less severe reaction if: cosensitization to PR‐10/profilin AD
Basagaña M et al. *J Investig Allergol Clin Immunol* 2018	Retrospective observational Children and adults	Spain	*N* = 84 F 64.3% Mean age 27.88 y (IQR, 3–62)	48.8% *Rosaceae* 28.6% tree nuts 7.1% other vegetables	94% Pru p 3 82.14% Jug r 3 76.19% Ara h 9 55.95% Cor a 8 16.6% Tri a 14	44% anaphylaxis 42.9% skin and/or oropharyngeal symptoms 13.1% asymptomatic sensitization to LTPs	41% cofactors	Lower mean Pru p 3 value in asymptomatic patients (*p* < .05) Sensitization to plane tree and mugwort more frequent in patients with food allergy Cofactors more present in patients with anaphylaxis The n of recognized LTPs in food‐allergic patients was greater than in asymptomatic patients, although the molecular spread did not affect the severity symptoms
Asero R et al. *Eur Ann Allergy Clin Immunol* 2018	Retrospective observational Children and adults	Italy	*N* = 67 F 53.7% Mean age 33.8 y (6–56)	71.6% *Rosaceae* 50.7% treenuts 19.4% peanut 19.4% tomato	44.8% LTP only Co‐sensitization: 28.3% PR‐10 7.5% profilin 19.4% both	52.2% S + L 25.4% S 22.4% L		No difference in Pru p 3 IgE levels between local vs. systemic reaction Monosensitized to LTP more at risk of systemic reaction Follow‐up (1–16 y): 27% new food allergies (2 anaphylaxis to pistachio, 1 sensitized to storage proteins) evolution did not depend total IgE level or co‐sensitization pattern
SLIT studies								
Navarro B et al. *Allergy Asthma Clin Immunol* 2019	Prospective, open label study Children and adults	Spain	*N* = 24 M 29.1% Mean age 25.5 (5–42) All with anaphylaxis to peach and sensitized to LTP	87% also anaphylaxis with nuts 12.5% also anaphylaxis with lettuce, plum and/or cherry			37.5% cofactors (4 Exercise and 5 NSAIDs)	Desensitization was induced in 79% of patients No severe reactions 29% mild oral symptoms, 8% urticaria associated with co‐factors 5 patients withdrew from study
Beitia JM et al. *Int Arch Allergy Immunol* 2021	Prospective, open label study Children and adults	Spain	*N* = 29 F 51.7% Median age 24.7 y (5.5–43.1) 5 children	100% *Rosaceae* 72%, peanut/nuts 27.5% other fruits/vegetables		19 had a history of severe systemic reactions 3 Grade 1 7 Grade 2		73% had negative OFC to peach at 1 year 95% had negative OFC to peach after 2 years 69% had negative OFC to nuts/peanuts Control group (*N* = 13): 53.8% experienced reactions with new foods; severity of symptoms increased significantly (*p* < .001), and diet restrictions were maintained in this group 7 patients discontinued therapy

Abbreviations: AD, Atopic dermatitis; AE, Angioedema; BAT, Basophil activation test; F, Female; GI, Gastrointestinal; IQR, Interquartile range; L, Local; LTP, Lipid transfer protein; N, Number; NSAIDs, Nonsteroidal anti‐inflammatory drugs; OAS, Oral allergy syndrome; OFC, Oral food challenge; PFA, Pollen‐food allergy; PR‐10, Pathogenesis‐related protein 10; R, Respiratory; S, Systemic; SPT, Skin prick test; SR, Systemic reaction; U, Urticaria; y, Years.

## 2 EPIDEMIOLOGY

### 2.1 Geography

The prevalence of LTP sensitization varies between geographical areas worldwide. Outside Europe, sensitization to LTP has not yet been reported in Africa,^1^ and there are few pediatric case reports from China,^12^ Australia,^13^ and the United States.^14^ A recent retrospective study described a cohort of 32 children with LTP allergy from Latin America (Chile).^15^ In contrast, LTP sensitization is widespread in Europe, with a clear difference between Northern and Southern European countries. Severe clinical manifestations induced by LTP are more frequently observed in the Mediterranean area, where the LTP syndrome represents a frequent type of food allergy.^9^, ^16^ However, in recent years, a few case reports on sensitization to LTP in children also emerged in other European regions outside the “endemic” Mediterranean area, such as the Netherlands,^17^ and France.^18^ For example, sensitization to the LTP Cor a 8 in hazelnut presents as a substantial risk factor for severe allergic signs and symptoms in children from these regions.^17^

In Spain, an epidemiological survey including both children and adults reported that around 50% of patients sensitized to Pru p 3 reported a food allergy.^5^, ^19^ These data were confirmed by another Spanish study reporting that 53 out of 430 outpatients (12.3%) older than 4 years seen in the allergy unit for any reason were sensitized to Pru p 3.^20^ Scala et al. reported that 9% of 23,000 adult and pediatric patients residing in Central and Southern Italy were sensitized to Pru p 3, detected by ISAC microarray.^21^ There are significant geographical variations in the prevalence of LTP sensitization even within individual countries, such as in Italy, where it is higher in the Central and Southern regions than in the Northern region.^22^

### 2.2 Sex

Sex differences may affect food allergy clinical manifestations, possibly due to changes in estrogen and progesterone levels that promote a Th2 response.^23^ In a study group of 26 children with LTP allergy, there was no difference in gender and severity of reactions.^9^ However, in a large Italian cohort of adults and children sensitized to Pru p 3, 70.9% of the patients were female, although specific percentages for the pediatric population alone are not available.^21^ A follow‐up study over 10 years revealed that individuals sensitized to LTP developed allergic signs and symptoms to plant foods they had previously tolerated, with a notably higher prevalence in female patients (60%).^24^ Furthermore, another study indicated that cofactor‐dependent LTP allergy is more common in women (>16 years old), which could be associated with menstruation.^25^

## 3 LTP STRUCTURE, ORIGIN, AND DISTRIBUTION

### 3.1 Allergen structure characterization

The primary function of LTPs is to facilitate lipid transfer between membranes by binding and solubilizing them.^26^ Additionally, LTPs are implicated in defending plants against bacterial and fungal pathogens,^27^, ^28^ categorizing them as pathogenesis‐related proteins of type 14 (PR‐14).^29^

LTPs are small, basic, non‐glycosylated proteins of about 6–9 kDa that share a highly conserved molecular structure with four alpha‐helices connected by disulfide bridges that impart resistance to heat and pH changes; these characteristics confer on LTP the ability to act as a primary sensitizer.^1^ The structure includes an internal hydrophobic tunnel‐like cavity that facilitates the transfer of various lipid molecules.^30^, ^31^, ^32^ Binding of lipids can modify the allergen's conformation, particularly affecting amino acids in the C‐terminal region. These structural changes can influence the allergen's surface and its capacity to bind IgE, as observed in Pru p 3, Jug r 3, and Mal d 3.^33^, ^34^, ^35^ LTPs are categorized into two subfamilies based on molecular weight: LTP1 (9–10 kDa) and LTP2 (6–7 kDa), with most allergenic LTPs being of the LTP1 type.^1^ For example, Pru p 3 belongs to the LTP1 type, while tomatoes have two types of LTP: Sola l 3 is LTP1, and Sola l 6 is LTP2.^36^, ^37^ To date, 59 LTPs are recognized by the WHO/IUIS Allergen Nomenclature Subcommittee^38^ (Table 2).

**TABLE 2:** LTP allergens in plant foods (A) and pollens (B) – as listed in the WHO/IUIS Allergen Nomenclature Database.

Species (common name)	Molecular allergens	Biochemical category
A. Foods		
*Actinidia chinensis* (gold kiwi fruit)	Act c 10	nsLTP1
*Actinidia deliciosa* (green kiwi fruit)	Act d 10	nsLTP1
*Apium graveolens* (celery)	Api g 2 Api g 6	nsLTP1 nsLTP2
*Arachis hypogaea* (peanut)	Ara h 9 Ara h 16 Ara h 17	nsLTP1 nsLTP2 nsLTP1
*Asparagus officinalis* (asparagus)	Aspa o 1	nsLTP1
*Brassica oleracea* (cabbage)	Bra o 3	nsLTP1
*Castanea sativa* (chestnut)	Cas s 8	nsLTP1
*Citrus limon* (lemon)	Cit l 3	nsLTP1
*Citrus reticulata* (tangerine)	Cit r 3	nsLTP1
*Citrus sinensis* (sweet orange)	Cit s 3	nsLTP1
*Corylus avellana* (hazelnut)	Cor a 8	nsLTP1
*Fragaria ananassa* (strawberry)	Fra a 3	nsLTP1
*Helianthus annuus* (sunflower seed)	Hel a 3	nsLTP1
*Juglans regia* (walnut)	Jug r 3 Jug r 8	nsLTP1 nsLTP2
*Lactuca sativa* (lettuce)	Lac s 1	nsLTP1
*Lens culinaris* (lentil)	Len c 3	nsLTP1
*Lupinus angustifolius* (narrow‐leaved blue lupin)	Lup an 3	nsLTP1
*Malus domestica* (apple)	Mal d 3	nsLTP1
*Morus nigra* (mulberry)	Mor n 3	nsLTP1
*Musa acuminata* (banana)	Mus a 3	nsLTP1
*Phaseolus vulgaris* (green bean, French bean)	Pha v 3	nsLTP1
*Pisum sativum* (pea)	Pis s 3	nsLTP1
*Prunus armeniaca* (apricot)	Pru ar 3	nsLTP1
*Prunus avium* (sweet cherry)	Pru av 3	nsLTP1
*Prunus domestica* (European plum)	Pru d 3	nsLTP1
*Prunus dulcis* (almond)	Pru du 3	nsLTP1
*Prunus persica* (peach)	Pru p 3	nsLTP1
*Punica granatum* (pomegranate)	Pun g 1	nsLTP1
*Pyrus communis* (pear)	Pyr c 3	nsLTP1
*Rubus idaeus* (red raspberry)	Rub i 3	nsLTP1
*Sinapis alba* (yellow mustard)	Sin a 3	nsLTP1
*Solanum lycopersicum* (tomato)	Sola l 3 Sola l 6 Sola l 7	nsLTP1 nsLTP2 nsLTP1
*Triticum aestivum* (wheat)	Tri a 14	nsLTP1
*Triticum turgidum ssp durum* (durum wheat)	Tri tu 14	nsLTP1
*Vitis vinifera* (grape)	Vit v 1	nsLTP1
*Zea mays* (maize)	Zea m 14	nsLTP1
B. Pollens		
*Ambrosia artemisiifolia* (short ragweed)	Amb a 6	nsLTP1
*Artemisia annua* (sweet wormwood)	Art an 3	nsLTP1
*Artemisia argyi* (silvery wormwood)	Art ar 3	nsLTP1
*Artemisia capillaris* (wormwood)	Art ca 3	nsLTP1
*Artemisia gmelinii* (Russian wormwood)	Art gm 3	nsLTP1
*Artemisia lavandulifolia* (mugwort)	Art la 3	nsLTP1
*Artemisia sieversiana* (Sieversian wormwood)	Art si 3	nsLTP1
*Artemisia vulgaris* (mugwort)	Art v 3	nsLTP1
*Cannabis sativa* (Indian hemp)	Can s 3	nsLTP1
*Hevea brasiliensis* (para rubber tree latex)	Hev b 12	nsLTP1
*Parietaria Judaica* (wall pellitory)	Par j 1 Par j 2	PhosphoLTP PhosphoLTP
*Parietaria officinalis* (pellitory)	Par o 1	PhosphoLTP
*Olea europaea* (olive tree)	Ole e 7	nsLTP1
*Platanus acerifolia* (London plane tree)	Pla a 3	nsLTP1
*Platanus orientalis* (oriental plane tree)	Pla or 3	nsLTP1
*Broussonetia papyrifera* (paper mulberry)	Bro p 3	nsLTP1

### 3.2 LTP containing foods

Peach is the most common trigger of LTP allergy in Southern Europe, alongside other *Rosaceae* fruits. Peanuts and tree nuts, especially walnuts and hazelnuts, are also common triggers. LTPs are present in a variety of other foods, including major cereals (wheat, maize, rice), as well as in green beans, fennel, oranges, kiwis, and lentils.^2^, ^29^

Triggers for LTP‐related allergic reactions in children are similar to those observed in the adult population.^2^ Pru p 3 is the major allergen for peach allergy in Spanish children.^39^, ^40^ In another study involving 130 Spanish children with a convincing history of immediate allergic reactions to plant foods and LTP sensitization, the foods most frequently causing reactions were peach (83.1%), walnut (77%), peanut (56.2%), and hazelnut (55.4%). A significant 69.2% had reactions to two or more taxonomically unrelated plant foods.^6^ These data are consistent with a Portuguese study on 26 children with LTP allergy, in which 69% reacted to more than one food. Fruits were involved in 69% of cases, with peach being the most frequent trigger (62%), followed by tree nuts (50%), whereas peanuts and sesame only affected 8% and 4%, respectively. During the follow‐up period, 12% experienced reactions to new LTP‐containing foods, occurring at various times, from less than 1 year to as long as 8 years. This finding highlights the possible evolving nature of LTP allergy in children, where new sensitizations to different LTP‐containing foods can develop over a broad time range.^9^

In a study examining 1271 Italian children aged 4–18 years with seasonal allergic rhino‐conjunctivitis, a cluster consisting of 36 children, mainly from Southern Italy, showed sensitivity to LTP (Pru p 3) and *Parietaria* pollen. These children typically developed seasonal allergic rhino‐conjunctivitis earlier in their lives and commonly had oral signs and symptoms induced by *Rosaceae*, banana, peanut, and hazelnut.^7^ In a Latin American cohort of children with LTP allergy, non‐*Rosaceae* family fruits, legumes, and tree nuts emerged as the most common allergens, followed by *Rosaceae* family fruits, vegetables, and seeds. Interestingly, among these, tree nuts (specifically walnut and almond) were more frequently associated with anaphylactic reactions, rather than peach.^15^ This variation in allergen prevalence and severity of reactions may be attributed to the distinct dietary patterns and eating habits prevalent in South America, but also to the inhalation of pollens of different allergenic plants.^41^ There have also been reports of less common LTP‐containing foods triggering allergic reactions in children, including garlic and onion,^42^ apple seed and grape,^13^ and blueberries.^12^ There were also case reports of wheat anaphylaxis related to wheat LTP (Tri a 14),^43^ and a case of allergy to barley LTP (Hor v 14).^18^

In a cohort of 57 children allergic to LTP, 37% reported reactions to processed foods, such as commercial peach juice, marmalade, canned peach, and commercial fruit puree.^40^ LTPs can also act as hidden allergens in various composite foods.^44^ For example, pizza with tomato sauce frequently causes allergic reactions in LTP‐allergic patients.^45^ LTP‐allergic patients may also show reactions to onion and garlic contained in many pre‐prepared and restaurant meals.^42^

### 3.3 Allergenic potency and distribution of LTP in foods

Typically, LTP is found in higher concentrations in the peel of fruits, with the inner pulp containing significantly lesser amounts.^1^ Carnes et al. assessed the concentration of LTP in peach extracts, finding that LTP levels in peel extracts were about seven times higher than in the pulp.^46^ In another study, a high concentration of LTP was also observed in the peel, which was 2.5 times greater than that in the pulp.^47^ A study on Spanish children with a history of reactions to peach showed that over 90% tolerated peach pulp, confirming that the primary allergen, Pru p 3, may be confined mainly to the peel or minimally present in the pulp.^40^

The concentration of LTP varies not only between different fruits but also among different cultivations and varieties of the same fruit.^48^, ^49^ For example, in the case of apple allergy in children, reactions to LTP (Mal d 3) were found to be more common and severe with Golden apple varieties as compared to the Stark or Smith varieties, indicating a variation in allergen content across different apple cultivars.^50^ High levels of LTP are also found in the seeds of fruits and vegetables, such as kiwi and tomato.^51^, ^52^, ^53^

Cooked and processed foods, including fruit or vegetables, can still trigger allergic reactions. This is due to LTPs being highly resistant to heat and digestive processes, and thus remain intact not just in raw foods but also in cooked ones.^2^

## 4 PATHOPHYSIOLOGY

### 4.1 Routes of sensitization

**FIGURE 1:**
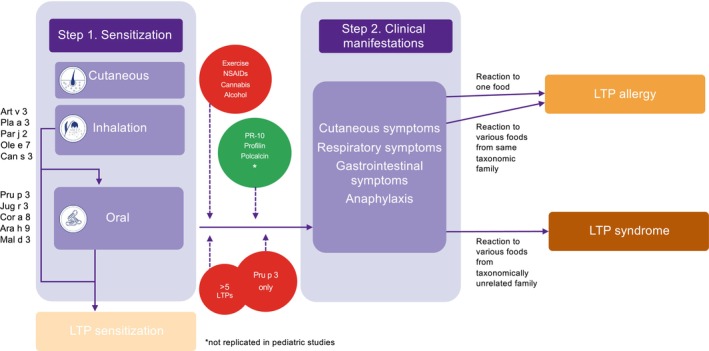
Routes of LTP allergen sensitization (Step 1) and possible attenuating (green) or aggravating (red) factors leading to development of clinical symptoms (Step 2), expressing as either LTP allergy or LTP syndrome. LTP, Lipid transfer protein; NSAIDs, Nonsteroidal anti‐inflammatory drugs; PR‐10, Pathogenesis‐related protein 10.

Sensitization to LTP is often present in early life^7^ and can occur in several ways, for example, cutaneous, gastrointestinal, or inhalation.^1^, ^54^ (Figure 1) Most of the available data come from studies conducted in adult populations, while the routes of LTP sensitization in children are less well known.^55^

In a study by Asero,^56^ the association between peach‐induced contact urticaria and sensitization to LTP in patients over 14 years old suggested a possible cutaneous sensitization route. However, the high capacity of Pru p 3 to penetrate the gastric barrier might also explain its potential to cause sensitization via this route.^57^ Sensitization to LTP in foods may also happen through inhalation. Studies have revealed that peach leaf extracts contain high levels of Pru p 3, comparable to those in peach peel, suggesting its potential role as a respiratory allergen leading to conditions like rhino‐conjunctivitis and asthma, preceding food allergy.^58^ Indeed, Pru p 3 has been identified as an occupational respiratory allergen among peach crop workers.^59^ Similarly, LTP in other foods, such as asparagus,^60^ maize,^61^ spelt,^62^ and wheat,^63^ has been noted for its significance in occupational allergies among vegetable food crop workers.

Other minor routes of sensitization, that is, through the nasal^64^ and oral mucosa,^65^ have been hypothesized. Sensitization to LTP following inhalation of Cannabis sativa has also been described in recent years.^66^ Two pediatric cases, involving 13‐ and 14‐year‐old patients, have been reported in which LTP from Cannabis sativa (Can s 3) was identified as the allergen responsible for anaphylactic reactions triggered by second‐hand exposure to Cannabis sativa cigarette smoke.^67^

### 4.2 Pollen LTP

Sensitization to LTPs in pollens plays a significant role in the development of food allergy in children. Early sensitization to LTPs from pollens such as plane tree and mugwort can lead to cross‐reactivity with food LTPs.^4^, ^68^ Table 2, part B, summarizes the LTPs that have been identified in plants to date.

In Mediterranean patients, peach is often identified as the likely primary sensitizer for LTP allergies, but this seems less probable in other geographical regions. In a study involving children sensitized to hazelnut from a non‐Mediterranean, birch‐endemic area, only a minority exhibited IgE antibodies to peach LTP (Pru p 3), with the majority having consumed peaches without experiencing allergic reactions.^17^ Considering the geographical disparities in LTP sensitization, it is speculated that the primary cause might be sensitization to pollen LTPs. In fact, LTPs have been found to be significant allergens in numerous types of tree and weed pollens, such as *Parietaria* (Par j 2), *Artemisia* (Art v 3), *Platanus* (Pla a 3), and *Olea* (Ole e 7).^29^ In a cohort of 130 children with LTP allergy, 69.3% were positive to pollen LTP, in particular 60.8% to Pla a 3, 50.8% to Art v 3, 24.6% to Ole e 7, and/or 14.6% to Par j 2. A strong association was also observed between sensitization to Cor a 8 and/or Ara h 9 and sensitization to the pollen‐LTPs Pla a 3 and Art v 3 (77.8%–90.4%). While 70% of pollen LTP‐sensitized patients reacted to all tested plant‐food LTPs, only 2.5% of non‐sensitized individuals did. Conversely, only 6.7% of pollen LTP‐sensitized subjects reacted to a single plant‐food LTP, compared to 57.5% in the non‐sensitized group (*p* < .001).^6^

There is conflicting evidence from Southern Europe that, in adults, sensitization to LTP pollens, such as plane tree (Pla a 3) and mugwort (Art v 3), may be related to food sensitization.^69^

*Parietaria* (Par j 2) and olive (Ole e 7) are other allergenic LTP pollens, although apparently of minor significance.^70^ Ciprandi et al. reported that in a group of Pru p 3‐positive children with Parietaria pollen allergic rhinitis, a quarter presented with anaphylaxis, and about half reported oral allergy signs and symptoms after eating foods containing LTP.^16^

### 4.3 LTP sensitization, LTP allergy, and LTP syndrome

The sensitization to LTP consists of the positivity of sIgE to one or more LTPs. When LTP sensitization is associated with reactions to one food or more from a single taxonomic group, it is called LTP allergy. In contrast, if it is associated with multiple reactions to various taxonomically unrelated foods, it is known as LTP syndrome. However, there is also the possibility of LTP sensitization without related allergic reactions. In these cases, the positivity of many cross‐reactive but clinically irrelevant sIgE makes the diagnosis more complex.^1^, ^2^ (Figure 1).

Many factors, such as age and geographic area, can influence the clinical relevance of these allergens, as well as the primary sensitizer.^71^ There is a hypothesis suggesting a hierarchy in the sensitization process to different LTPs,^54^ typically starting with peach and progressing to other *Rosaceae* fruits, then tree nuts like hazelnut and walnut, and less frequently to lentil, maize, soybean, tomato, kiwi, sesame, mustard, melon, and celery. This pattern appears to be linked to Pru p 3 sIgE levels: higher peach sIgE levels increase the likelihood of positive tests for other plant‐based foods.^71^ In contrast, patients with low Pru p 3 IgE levels rarely recognize LTP from other food sources.^72^ In a study of 426 Italian LTP allergy patients aged 2–74 years, 17.8% showed reactivity to a single LTP molecule, with 46.1% responding to Pru p 3. Additionally, 45.3% were sensitized to up to four molecules. Patients tolerating LTP sources had notably lower IgE values toward these molecules than non‐tolerant individuals. Furthermore, the recognition of more than 5 LTPs was significantly associated with an increased risk of severe reactions.^73^ In a study of eastern Mediterranean children with multiple sensitizations, 21% were found to have LTP sensitization. The most common sensitizations were to Pru p 3 (74%) and Cor a 8 (66%). Clinical reactivity, reported in almost half of the children, was associated with increasing age and the number of LTP molecules they were sensitized to.^74^

The similarities and differences in amino acid sequences, protein structures, and IgE epitopes among LTPs may explain patient clinical presentations. Cross‐reactivity between homologous molecules depends largely on the percentage of amino acid identity: it is unlikely below 50% but highly probable above 70%. In a study by Scala et al.,^73^ Pru p 3 showed high sequence identity with taxonomically related LTPs, such as Pyr c 3 (pear) and Pru d 3 (plum), while LTP2 molecules like Api g 6 (celery) and Sola l 6 (tomato) also display significant identity with other LTP2s, such as Ara h 16 (peanut). IgE co‐recognition was strongest among molecules with the highest amino acid sequence identity, with significant associations observed between Mal d 3 and molecules such as Ara h 9, Cor a 8, Jug r 3, Pla a 3, and Pru p 3. In another study by the same group,^75^ 568 nsLTP‐positive children and adults were assessed using ImmunoCAP‐ISAC to analyze sensitization patterns and clinical phenotypes. The patients clustered into different groups according to the sensitization profile. Sensitization to Art v 3 and Pla a 3 was significantly associated with rhinoconjunctivitis, while Pru p 3‐positive subjects had a lower frequency of respiratory allergy. Pru p 3 was the main sensitizer in children <6 years, while in patients >15 years, Jug r 3 reached comparable levels of reactivity, indicating walnut as an alternative sensitizer in older populations. Pru p 3 and Pla a 3 sensitization were strongly correlated, whereas Tri a 14 showed a weaker correlation, not fully explained by comparing amino acid identity. The question of which acts as a primary sensitizer remains unresolved: Art v 3 and Pla a 3 primary sensitization may trigger epitope spreading and enhance food LTP reactivity, but it cannot be excluded that sensitization to pollen LTP is the result of food LTPs cross‐reactivity. Longitudinal studies are needed to investigate the mechanism of co‐sensitization to pollen and food LTPs and its influence on the clinical spectrum, even more so in children.

## 5 CLINICAL MANIFESTATIONS

### 5.1 Signs and symptoms

Children diagnosed with LTP allergy, similar to adults, exhibit a highly diverse range of signs and symptoms, including urticaria, oropharyngeal clinical manifestations, angioedema, respiratory issues, gastrointestinal clinical manifestations, and even anaphylaxis.^2^, ^3^, ^54^ The number of allergic episodes among children can vary: in a study including 57 Spanish children allergic to peach, 30% experienced one reaction, 37% experienced two reactions, and 26% experienced three or more reactions.^40^

In a study comprising adults and children ranging in age from 3 to 62 years old and sensitized to Pru p 3, 44% experienced anaphylaxis, 43% had skin or oropharyngeal reactions, and 13% remained asymptomatic.^76^ In a study of 82 Italian children allergic to *Parietaria* pollen and sensitized to Pru p 3, one‐quarter experienced anaphylaxis, while half had milder reactions. Compared to 29 adults from the same geographic area and with the same sensitization profile, reactions were more common in children (73.2% vs. 37.3%), with anaphylaxis occurring only in children. Conversely, three‐quarters of adults reported oral allergy syndrome, compared to just one‐quarter of children.^16^ In a study on 26 Portuguese children with LTP allergy, the signs and symptoms observed included urticaria in 58%, anaphylaxis in 46%, and oral allergy syndrome in 42% of the participants.^9^ Another study has reported similar findings on 130 Spanish children sensitized to LTP.^6^ Latin American pediatric patients with LTP allergy showed a higher anaphylaxis rate (53%)^15^ compared to European children (30%–40%).^9^, ^25^ This could be attributed to different eating habits, as also evidenced by the different frequencies of LTP‐containing food triggers, with a high prevalence of tree nuts.^15^ In fact, fruits and vegetables are less frequently reported as anaphylaxis triggers than nuts, seeds, and legumes.^6^

### 5.2 Influence of PR‐10 and Profilins on the clinical severity of LTP allergy

Some studies indicate that being co‐sensitized to PR‐10 allergens, such as Bet v 1 from *Betula verrucosa* and/or to profilin, might act as a protective factor against the severity of LTP allergic reactions.^77^, ^78^ However, among the various studies on the pediatric population with LTP allergy, few assess the impact of co‐sensitization to PR‐10 and profilins on the severity of signs and symptoms. Recent studies specifically targeting the LTP allergic pediatric group have not identified any association between co‐sensitization to PR‐10 or profilins and a decrease in the severity of clinical manifestations.^9^, ^15^ In particular, in the study focusing on a group of South American children with LTP allergy, a high incidence of anaphylaxis is reported among patients co‐sensitized to profilins, which contrasts with findings from European reports for adult patients.^15^, ^41^

### 5.3 Cofactors

The clinical expression of LTP sensitization often depends on the presence of cofactors, which are present in up to one‐third of allergic reactions to foods containing LTPs. Therefore, patients with cofactor‐induced allergic reactions should be able to eat culprit foods without cofactors without presenting symptoms. The best‐known cofactors include physical exercise (FDEIA, food‐dependent exercise‐induced anaphylaxis), non‐steroidal anti‐inflammatory drugs/NSAIDs (FDNIH, food‐dependent NSAID‐induced hypersensitivity) and alcohol, but many others have been investigated (e.g., sleep deprivation, oral mucosal lesions, fasting, concurrent viral infection, antacids, bariatric surgery, estrogens).^2^, ^3^, ^79^ Cofactor presence can also be associated with an increase in reaction severity. The underlying mechanisms of cofactors in allergic responses remain incompletely elucidated. With regard to physical exercise, it is hypothesized that it may involve an increase in gut permeability, changes in plasma osmolarity, blood flow redistribution, basophil and mast cell activity, diamine oxidase inhibition, and metabolism of eicosanoids and adenosine.^80^, ^81^

Like in adults, cofactors also play a significant role in triggering allergic reactions to the ingestion of LTP‐containing foods in children. In a group of patients, encompassing both children and adults, 10.8% had a history of FDEIA, and 12.4% experienced FDNIH, with most of these patients showing reactivity to over five LTP molecules.^73^ In a cohort of Spanish children with LTP allergy, cofactors (exercise and NSAIDs) were reported in 27% of cases, and most had more severe reactions in their presence. In some cases, cofactors proved necessary for the occurrence of reactions.^9^ Mota et al. reported three young patients (respectively, 11, 16, and 18 years old) with FDEIA in a group of 11 LTP allergic patients under 18 years old.^82^

However, Pascal et al.^6^ noted a lesser involvement of cofactors, with only three out of 130 LTP‐sensitized children (aged 8, 11, and 14, respectively) experiencing FDEIA, resulting in a total of five reactions. It's important to note that this discrepancy could be attributed to difficulty in identifying cofactors in children. Parents may overlook or not mention occurrences of physical activity, such as running or jumping, or intense emotional responses, like crying or excitement, considering them as typical aspects of a child's daily life. Although the most crucial allergen in wheat‐dependent exercise‐induced anaphylaxis (WDEIA) is omega‐5 gliadin (Tri a 19), cases have been reported in which the culprit allergen was wheat LTP Tri a 14.^83^ In recent years, there have also been reports of pediatric cases of WDEIA triggered by LTP.^84^, ^85^

## 6 DIAGNOSIS

### 6.1 Clinical history

As recommended by the *2023 EAACI guidelines on the diagnosis of IgE‐mediated food allergy* ^86^ a comprehensive, allergy‐focused clinical history is the first recommended step in the diagnostic process for patients suspected of having LTP allergy. A detailed clinical history is crucial in selecting the appropriate tests for confirming LTP allergy and distinguishing it from other plant food allergies.^87^ Many children may be without clinical manifestations despite being sensitized to LTP. Therefore, screening for LTP should be based on specific clinical indications to avoid unnecessary dietary restrictions. The history should detail the suspected foods, the timing and severity of signs and symptoms, the consistency of reactions, the quantity and preparation of the food (such as whether it's raw or cooked, peeled or unpeeled), any relevant cofactors, and the severity of the reaction (i.e., adrenaline necessity). Additionally, assessing for allergic rhinitis is important to determine if reactions to plant foods might stem from cross‐reactions with pollen allergens like PR‐10 and profilin.^87^, ^88^, ^89^, ^90^

### 6.2 Skin Prick Test (SPT)

In children, Skin Prick Tests (SPTs) are often the first line of testing.^91^ The prick‐to‐prick method using fresh fruits and vegetables is generally more sensitive than SPTs with commercial extracts,^92^ but this method lacks standardization since the LTP content in fruits and vegetables can vary.^2^, ^93^ The selection of foods to be tested should be guided by the specific plant‐based foods implicated in the allergic reaction.^2^

A positive skin test result, whether with fresh food or standard commercial reagents, merely indicates sensitization and does not conclusively prove that LTP is the allergen responsible for the reaction.^19^, ^94^ In a study on LTP allergic children, there was no association between tolerance to *Rosaceae* family fruits, average SPT wheal size with peach LTP extract, number of positive fruits SPT, and Pru p 3 sIgE levels. Notably, SPTs with food extracts are not always reliable for confirming clinical reactivity, as evidenced by 38% of children showing positive SPTs to foods they later tolerated.^9^ Additionally, a negative test does not exclude LTP sensitization, as Pru p 3 does not fully represent all LTP sensitization profiles.^95^

### 6.3 Specific IgE and component‐resolved diagnosis (CRD)

Component‐Resolved Diagnosis (CRD) involves testing sIgE against individual molecular components of food allergens and has proven to be more specific compared to measuring sIgE against whole extracts.^2^, ^29^, ^86^ Pru p 3 is considered the most reliable biomarker for LTP sensitization, but it might not detect all patients due to possible sensitization to LTP from different sources.^96^

The level of sIgE to LTP, especially Pru p 3, might correlate with the severity of signs and symptoms and the risk of allergic reactions, although the evidence is conflicting for both adult^11^, ^95^, ^97^, ^98^ and pediatric populations.^6^, ^9^, ^16^, ^76^, ^99^ On the other hand, patients with very high IgE levels may still not have any reactions.^54^ Novembre et al.^99^ found that sIgE levels to Pru p 3 were not linked to systemic signs and symptoms, nor did they associate with the severity of reactions in children. A different study involving 26 children with LTP allergy supported this finding.^9^ Conversely, in a group of children with LTP allergy and sensitization to *Parietaria* pollen, Pru p 3 sIgE levels appeared to increase in relation to the severity of the reaction, being lower in children with oral allergy syndrome and higher in those experiencing anaphylaxis.^16^ In a different study, patients sensitized to LTP were categorized into three groups according to their reactions to plant foods, and it was found that those who were asymptomatic had a lower mean Pru p 3 value compared to the other groups, which included patients with anaphylaxis and skin/oral clinical manifestations.^76^

Moreover, high Pru p 3 sIgE levels have been linked to a greater likelihood of allergy to hazelnuts, peanuts, and tree nuts.^77^, ^97^, ^100^ A recent study on LTP allergy found that Ara h 9, Cor a 8, and Mal d 3 had the most robust links to clinical severity. These allergens were rarely positive in subjects who were tolerant (with 15%, 9%, and 20%, respectively); thus, the lack of such specific IgE reactivity strongly indicates tolerance to the particular allergen source.^73^

Nowadays, multiplex tests are also available as second‐ or third‐line investigations for LTP‐allergic patients, since they both allow the identification of multiple LTP sensitizations with a single exam, even though with slightly different sensitivity for the different molecules.^101^ However, these tests should be prescribed and interpreted by an experienced physician, since multiple sensitization does not necessarily imply multiple clinical reactivity.

The Basophil Activation Test (BAT) has been suggested as a tool to aid in the diagnosis of food allergies. However, limited evidence supports its effectiveness specifically for LTP allergy, and it is not widely available in all laboratories.^2^, ^86^, ^102^

### 6.4 Oral Food Challenge (OFC)

Whilst an oral food challenge (OFC) is considered the gold standard for confirming a food allergy diagnosis,^86^ for the purposes of diagnosing LTP allergy, an OFC cannot determine whether reactions are specifically due to sensitization to LTP. However, OFC may be useful for identifying tolerated foods and minimizing unnecessary dietary restrictions. In clinical settings, OFC faces several issues, including the absence of established threshold doses for plant foods and potential false negatives caused by low representation of LTPs in the food under test. In order to evaluate the role of cofactors, the OFC can be conducted before or after physical activity or administering aspirin.^2^, ^29^, ^103^, ^104^ In fact, a positive food and exercise challenge confirms the diagnosis, while a negative challenge does not exclude it.^105^, ^106^ In children with suspected FDEIA, when food and exercise alone do not trigger symptoms, adding aspirin to the challenge protocol helps reduce false‐negative results, increasing the diagnostic power.^107^, ^108^

## 7 MANAGEMENT

**FIGURE 2:**
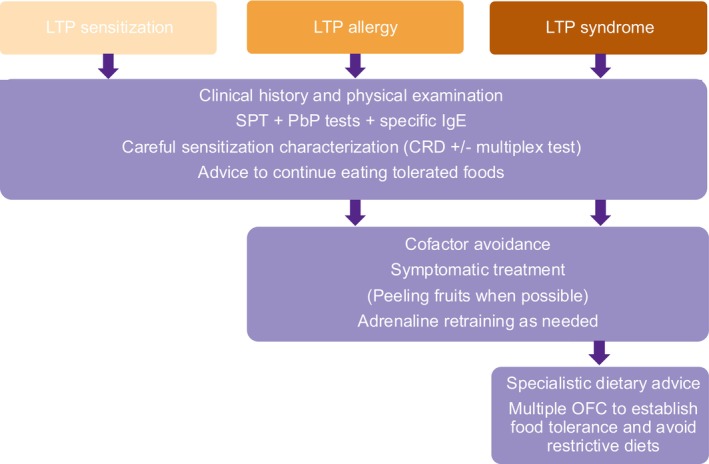
Flowchart of proposed management algorithm for LTP sensitization, LTP allergy and LTP syndrome. CRD, Component‐resolved diagnosis; LTP, Lipid transfer protein; PbP, Prick‐by‐prick; OFC, Oral food challenge; SPT, Skin prick test.

The initial approach of food allergy management involves removing the specific food that triggers the allergic reaction. However, in the case of LTP allergy, it becomes essential to provide carefully tailored dietary advice based on the patient's medical history.^2^, ^87^, ^109^ Figure 2 provides a summary of LTP allergy management.

Considering the prevalence of LTP across the plant kingdom and their high cross‐reactivity, a feasible approach for LTP allergic patients might be to continue consuming LTP‐containing foods they have previously tolerated rather than strictly avoiding these foods, which could lead to excluding almost all plant‐based foods from their diet. From an immunological point of view, especially in children, it is a reasonable practice that could contribute to maintaining a natural state of tolerance. Conversely, avoiding foods that are tolerated could lead to the development of an actual clinical allergy due to disrupted immunological tolerance resulting from a lack of allergen exposure.^110^, ^111^ In a study of 67 LTP‐allergic patients (age range 6–56 years), 27% developed new food allergies over a follow‐up of 1–16 years. Most new reactions involved *Rosaceae/Prunoideae*, tree nuts, and peanuts, with over half being systemic.^112^

Patients are often advised to take specific precautions: avoid combining LTP‐containing foods with cofactors like physical activity, NSAIDs, and alcohol, limit intake within a single meal or day, and peel fruits when possible. In pediatric cases, many children can tolerate fruits from the *Rosaceae* family when peeled, as LTPs are mainly present in the peels. It is essential to educate children and their caregivers on recognizing potential triggers, timely reaction identification, proper treatment, and the influence of cofactors in LTP allergies.^9^, ^110^, ^111^, ^112^ Besides dietary management, patients must have access to emergency medications, such as oral antihistamines and, when considered appropriate, self‐injected adrenaline, in accordance with current guidelines.^2^, ^113^

In recent years, immunotherapy has emerged as a promising treatment for food allergies, notably including Pru p 3 immunotherapy. A recent systematic review has indicated the effectiveness and favorable safety profile of Pru p 3 immunotherapy, showing positive outcomes in treating peach and LTP allergy for other foods, such as peanuts, although only a few studies included a pediatric population.^114^ A real‐life prospective study involved 29 adults and 5 children diagnosed with LTP allergy who were treated with Pru p 3 sublingual immunotherapy (SLIT). After 1 year of SLIT, 73% of the patients had a negative OFC to peach, which increased to 95% after 2 years, and 69% had a negative OFC to nuts/peanuts. In contrast, the control group, consisting of 13 patients with LTP syndrome, saw 53.8% experiencing reactions to new foods, with a significant increase in the severity of their signs and symptoms.^115^ A different approach was undertaken in a study where commercial peach juice served as oral immunotherapy (OIT) for 24 patients aged 5–42 years with a history of anaphylaxis in the previous 3 months. This treatment led to 79% of them successfully passing their OFCs after an average duration of 3.6 months.^116^ A recent protocol proposed the combination of Pru p 3 SLIT followed by OIT with peach juice after 40 days of the SLIT maintenance phase. The final OFC was successful in 39 of 45 patients aged 16 years and older (86.6%). One month post‐final provocation, 93.3% of the patients had no dietary restrictions, and there was a significant improvement in the quality of life.^117^ Pru p 3 immunotherapy is a promising treatment, yet its long‐term efficacy post‐discontinuation and effectiveness against cofactors are still unclear. Currently available only in Spain, its applicability could be limited to a specific population, where peach is believed to be the primary LTP sensitizer, while its effectiveness in other countries, where Pru p 3 might not be the primary sensitizing allergen, remains unknown.^2^

## 8 CONCLUSION

LTP allergy is more common in adults, especially in Southern Europe, but it is increasingly recognized also in children. The only study directly comparing age groups reported more severe reactions at a younger age, with adults experiencing milder symptoms, such as oral allergy syndrome.^16^ Children are also more frequently polysensitized to foods like *Rosaceae* fruits and nuts compared to adults. Cofactors like exercise or NSAID use affect both groups, but diagnosing children can be harder due to unrecognized triggers.

Management strategies focus on personalized dietary plans and emergency medications. Clinical practice varies due to the complexity of diagnosing and managing LTP allergy, with more research needed in children, as most studies focus on adults. Future diagnostic tools must better differentiate LTP sensitivities, and research should aim to standardize testing methods, such as the BAT, for more accurate diagnosis. The role of co‐sensitization with PR‐10 and profilins also requires further investigation.

While numerous studies provide evidence of LTP sensitization, fewer address its progression to clinically relevant allergy, especially across different foods. Differentiating between sensitization that leads to allergic symptoms and asymptomatic sensitization remains a key challenge for improving clinical practice.

Exploring the potential for natural tolerance, particularly through controlled exposure to tolerated foods, could improve long‐term management, especially for children. It is not yet clear whether the early introduction of LTP‐containing foods, including peach, may play a role in preventing or mitigating LTP allergy, but this approach could represent an area for further investigation. Education for patients, parents, and caregivers is critical for managing allergies and preventing severe reactions. Advancements in understanding LTP allergy, along with the potential expansion of LTP immunotherapy, offer hope for better, more patient‐friendly treatment options.

## AUTHOR CONTRIBUTIONS

Bianca Olivieri: conceptualization (lead); writing—original draft (lead); writing—review and editing (equal); visualization (equal). Mattia Giovannini: Conceptualization (lead), writing—original draft (lead); writing—review and editing (equal); visualization (equal). Benedetta Pessina: visualization (lead); writing—original draft (equal); writing—review and editing (equal). George du Toit: supervision (supporting); writing—review and editing (equal). Simona Barni: supervision (supporting); writing—review and editing (equal). Patrizia Bonadonna: supervision (supporting); writing—review and editing (equal). Marco Caminati: supervision (supporting); writing— review and editing (equal). Ru‐Xin Foong: writing—review and editing (equal). Francesca Mori: supervision (supporting); writing—review and editing (equal). Elio Novembre: supervision (supporting); writing—review and editing (equal). Gianenrico Senna: supervision (supporting); writing—review and editing (equal). Isabel Skypala: project administration (lead); supervision (supporting); writing—original draft (equal); writing—review and editing (equal).

## FUNDING INFORMATION

This research did not receive any specific grant from funding agencies in the public, commercial, or nonprofit sectors.

## CONFLICT OF INTEREST STATEMENT

MG reports personal fees from Sanofi. SB reports personal fees from Nutricia and Sanofi. All other authors declare that they have no conflicts of interest to disclose in relation to this paper.
